# Supplementary figures and images for: N6-methyladenosine RNA modification promotes Severe Fever with Thrombocytopenia Syndrome Virus infection
Source: PLoS Pathog. 2024 Nov 25;20(11):e1012725. doi: 10.1371/journal.ppat.1012725 (PMC11627400; doi:10.1371/journal.ppat.1012725)

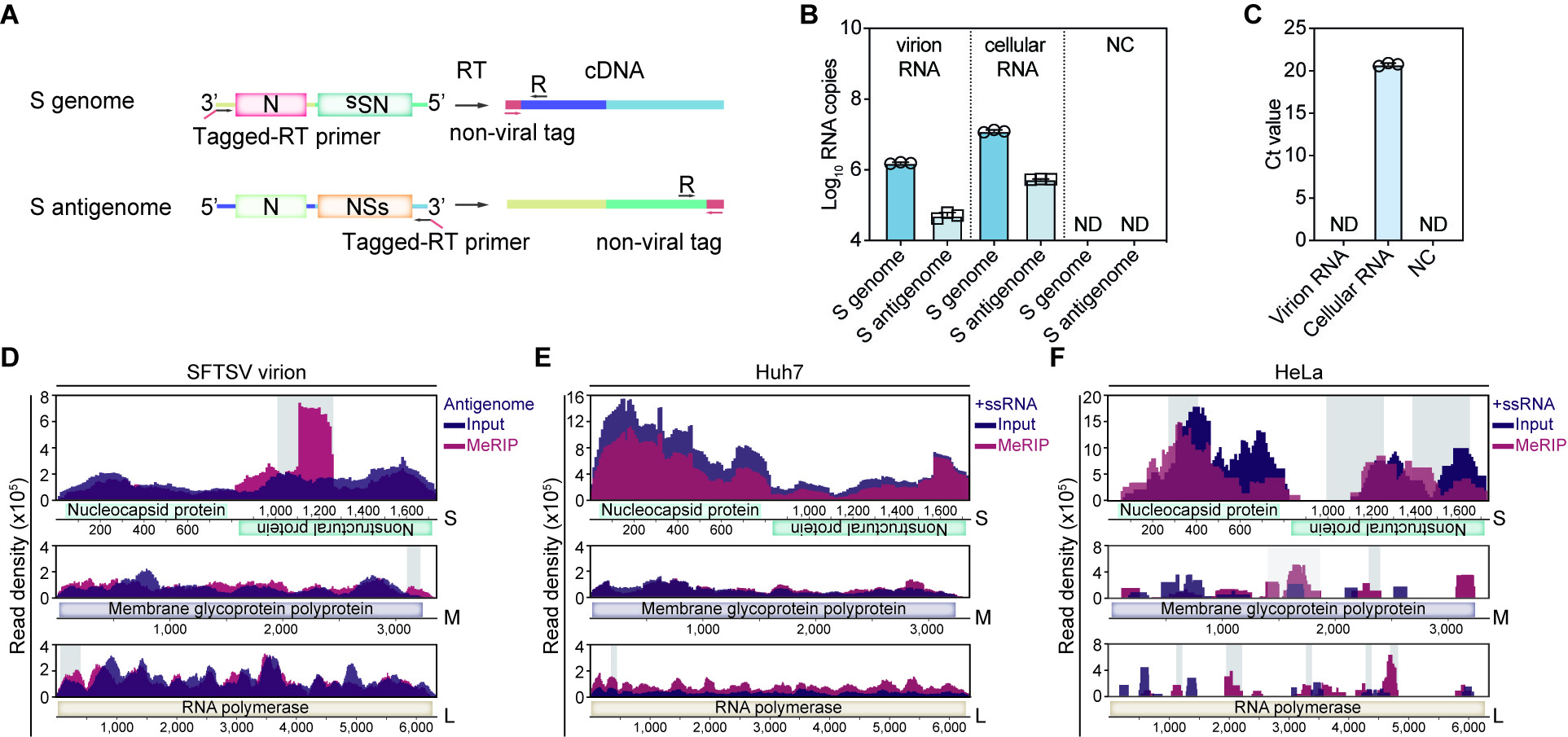

Supplement: S1 Fig — (A) Schematic diagram of strand-specific RT-qPCR. The strand-specific tagged reverse transcription primer (Tagged-RT primer) carrying a non-viral tag at the 5’ end is reverse complementary to the 3’-UTR region in the genome or antigenome. The qPCR uses a non-viral tag as forward primers, and strand-specific reverse primers are designed on the genome or antigenome. Using the plasmid encoding the SFTSV S genome as a template, the RNA of the S genome or antigenome was in-vitro transcribed to make the corresponding standard curve. (B) Stranded-specific RT-qPCR detection of the copy numbers of SFTSV S genome/antigenome. The total RNA isolated from Huh7 cells infected with SFTSV for 48 h was used as a positive control, and ddH2O was used as a negative control (NC). (C) RT-qPCR detection of ACTB mRNA with total RNA from (B) as a positive control and ddH2O as the NC. ACTB mRNA was not detectable in cDNA reversely transcribed from virion RNA using Oligo d(T). Data are representative of three independent experiments and presented as mean ± SD. (D-F) The distribution of m6A peaks in the S, M and L segments of SFTSV plus-sense RNA (+ssRNA). MeRIP-seq mapping of SFTSV RNAs derived from either purified virions (D) or SFTSV-infected Huh7 (E) or HeLa (F) cells. Antigenome/+ssRNA: The baseline signal of Input was displayed as blue, and the m6A IP signal was displayed as red. The gray rectangle indicated the m6A peaks recognized by MACS2 [45]. (TIF) [file ppat.1012725.s001.tif]

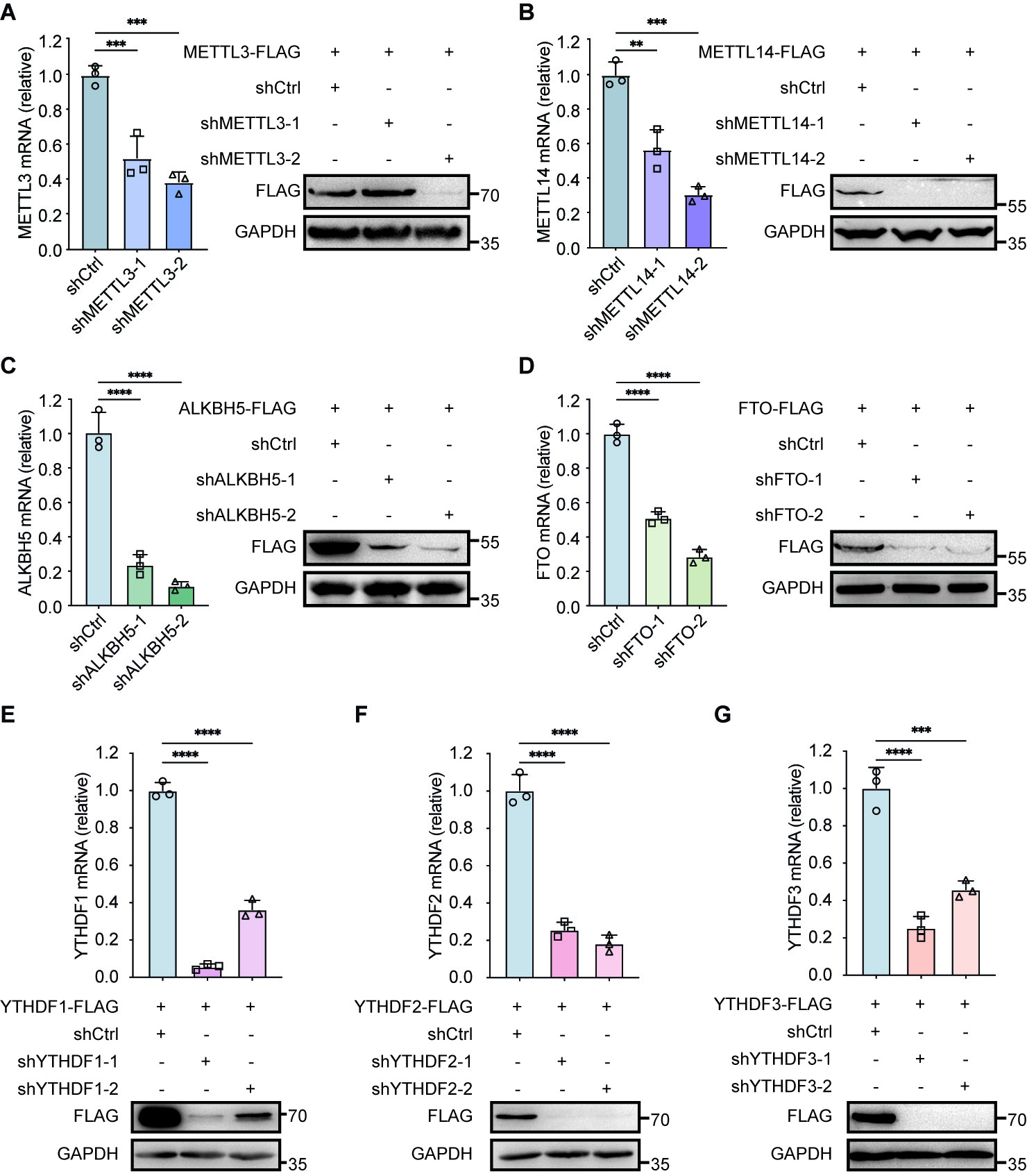

Supplement: S2 Fig — HEK293T cells were co-transfected FLAG-tagged m6A methyltransferases (METTL3 in A, and METTL14 in B), demethylases (ALKBH5 in C, and FTO in D) or readers (YTHDF1/2/3 in E-G, respectively) together with a control (shCtrl) or two different shRNAs targeting the overexpressed protein. mRNA and protein levels of each overexpressed molecule were measured by RT-qPCR with ACTB as the reference gene and by WB with GAPDH as the loading control, respectively. Data are representative of three independent experiments and presented as mean ± SD. Statistical significance was determined by one-way ANOVA followed by Dunett’s multiple comparisons test. **, P < 0.01; ***, P < 0.001; ****, P < 0.0001. (TIF) [file ppat.1012725.s002.tif]

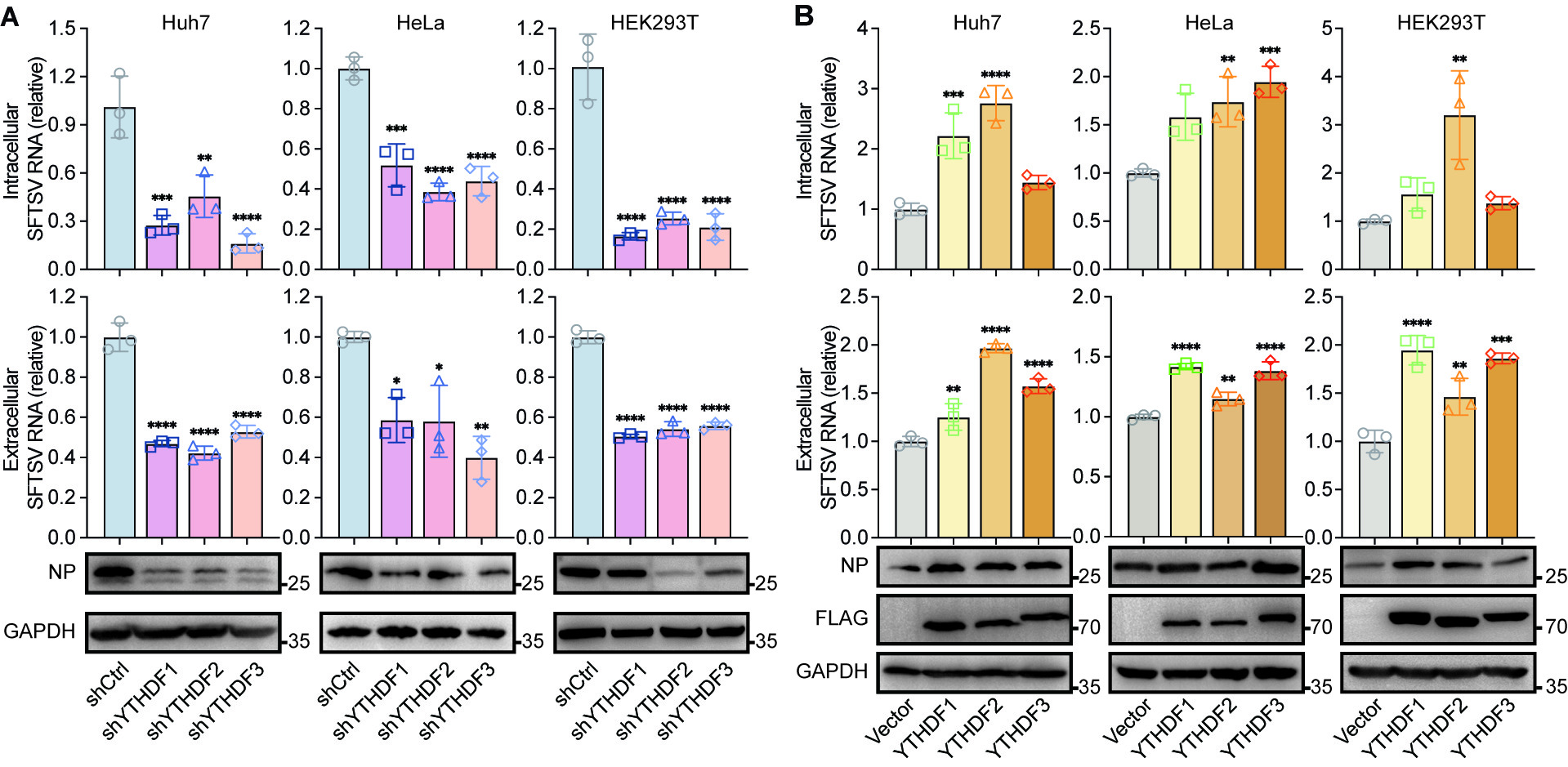

Supplement: S3 Fig — (A) Knockdown of YTHDF1/2/3 inhibits SFTSV infection. Huh7, HeLa or HEK293T cells were infected with lentiviral particles containing either control (shCtrl) or m6A readers-specific (shYTHDF1/2/3) shRNAs and selected with puromycin. (B) Overexpression of YTHDF1/2/3 promotes SFTSV infection. Huh7, HeLa, or HEK293T cells were transfected with plasmids encoding YTHDF1/2/3. After 24 h, cells were infected with SFTSV (MOI = 1) for 48 h. Relative cellular SFTSV RNA were quantified by RT-qPCR at 48 hpi with ACTB as the reference gene, while the released SFTSV RNA isolated from equal volumes of supernatants were quantified by RT-qPCR and normalized to the control group. Total cell extracts harvested at 48 hpi were subjected to western blot using antibody against SFTSV NP protein with GAPDH as a loading control. Data are representative of three independent experiments and presented as mean ± SD. Statistical significance was determined by one-way ANOVA followed by Dunett’s multiple comparisons test. *, P < 0.05; **, P < 0.01; ***, P < 0.001; ****, P < 0.0001. (TIF) [file ppat.1012725.s003.tif]

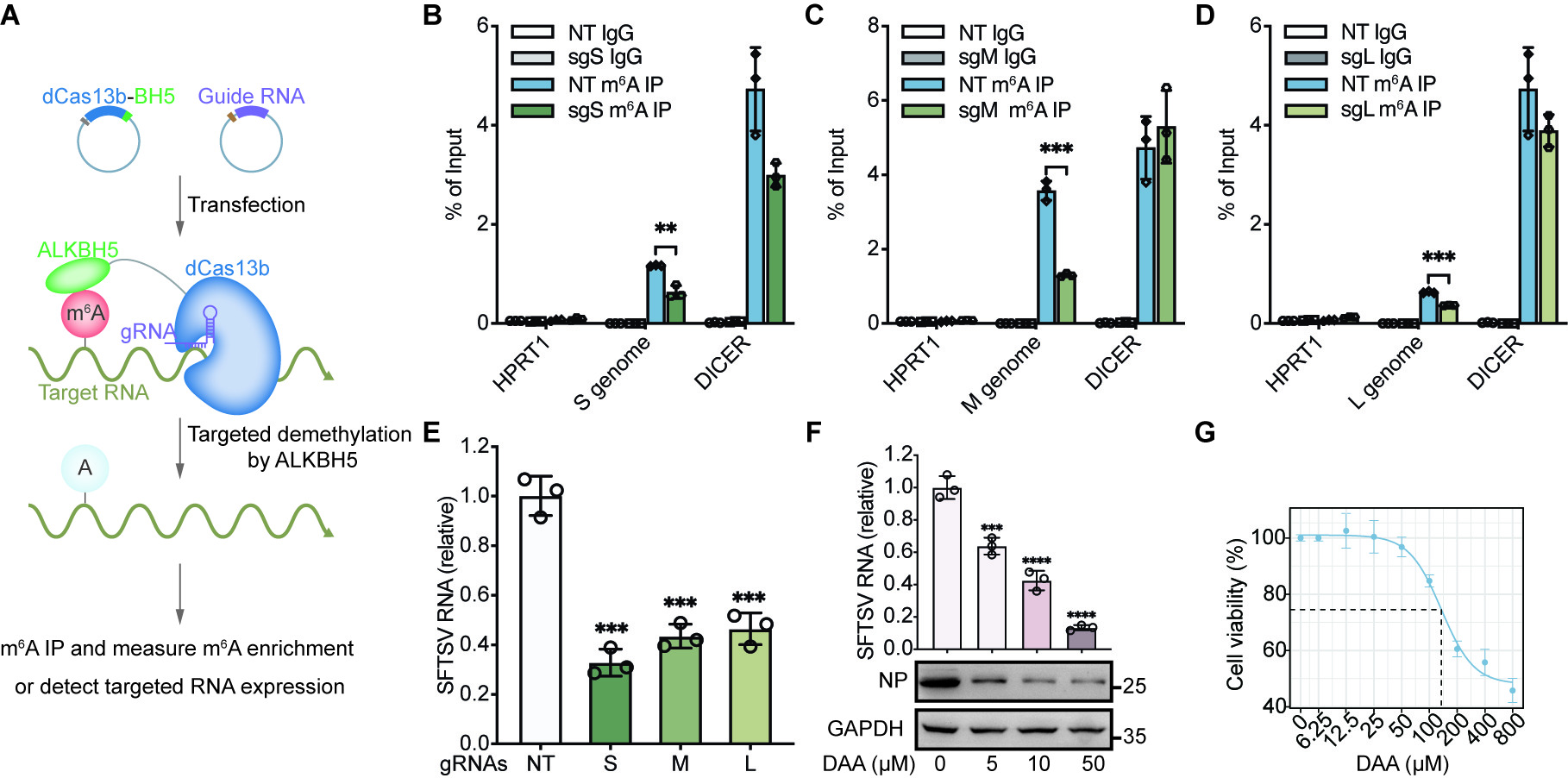

Supplement: S4 Fig — (A) Schematic of dCas13-ALKBH5 targeting assay. SFTSV-infected Huh7 cells were transfected with plasmids encoding dCas13b-ALKBH5 fusion protein and dCas13 guide RNAs targeting different SFTSV genomic RNAs. (B-D) m6A modification levels on S (B), M (C), and L (D) genome RNAs were determined by m6A-IP followed by RT-qPCR analysis, with HPRT1/DICER as negative/positive controls, respectively. (E) Expressions of SFTSV RNAs in SFTSV-infected Huh7 cells, which had been transfected with dCas13b-ALKBH5 and different gRNAs to target different sites of the viral genome RNA, was quantified by RT-qPCR. (F) The methylation inhibitor DAA restricts SFTSV infection. RT-qPCR analysis of SFTSV RNA expression or western blot analysis showing NP protein levels in DAA-treated and SFTSV-infected (MOI = 1) Huh7 cells at 48 hpi. (G) Cell viabilities of Huh7 cells after 48 h DAA treatment were assessed by the MTT assay. Data are representative of three independent experiments and presented as mean ± SD. Statistical significance was determined by two-way ANOVA followed by Sidak’s multiple comparisons test (B-D), or one-way ANOVA followed by Dunett’s multiple comparisons test (E and F). **, P < 0.01; ***, P < 0.001; ****, P < 0.0001. (TIF) [file ppat.1012725.s004.tif]

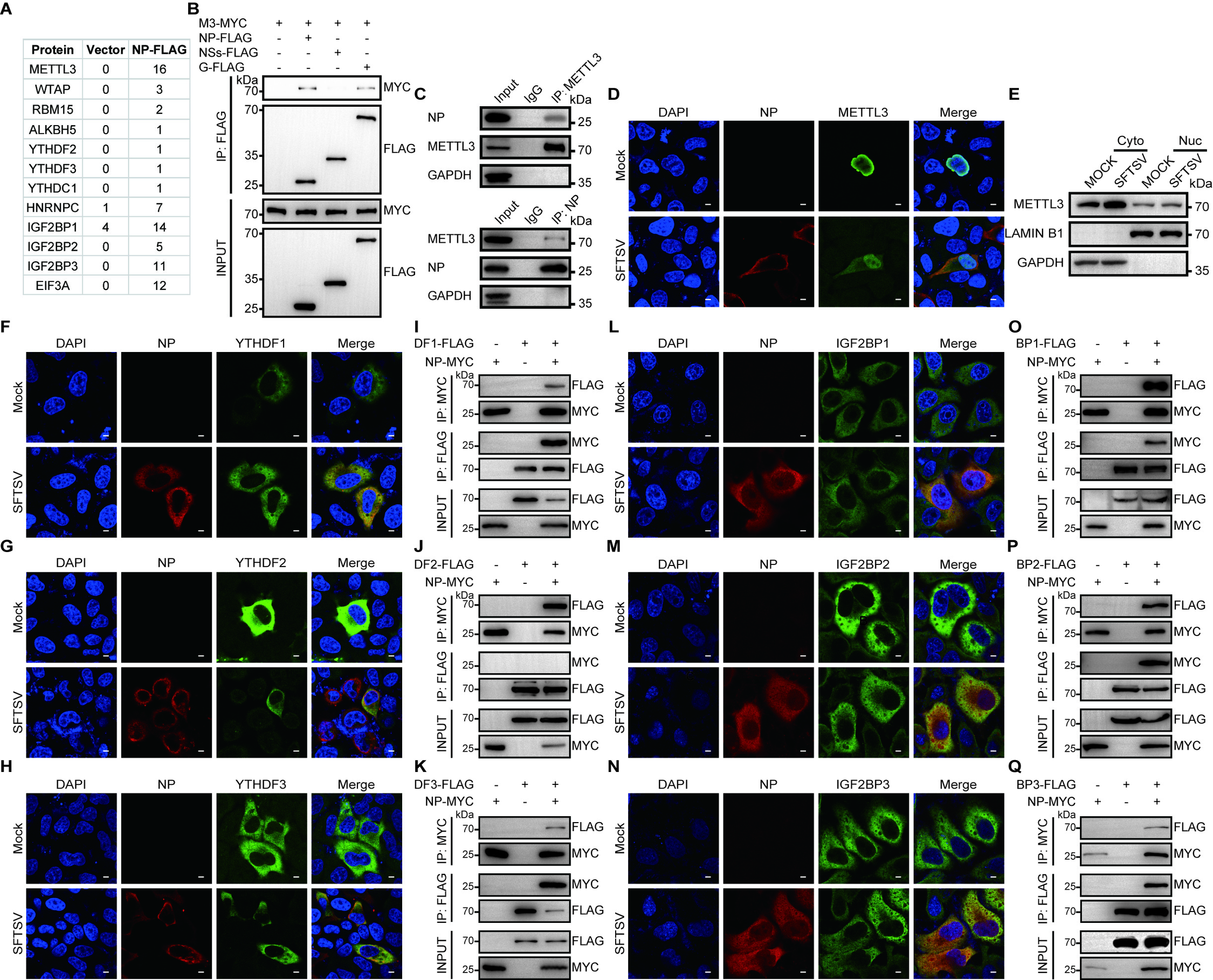

Supplement: S5 Fig — (A) Potential NP-interacting m6A regulators identified by IP-MS analysis. (B) Co-IP of METTL3 with several SFTSV proteins. HEK293T cells were co-transfected with Myc-tagged METTL3 plasmid and each Flag-tagged NP/NSs/G plasmid. At 36 h post transfection, Co-IP was performed by incubating the cell lysates with the anti-Flag antibody-coupled magnetic beads overnight, and the resulting immunoprecipitates were analyzed by western blotting with indicated antibodies. (C) Co-IP assay of the endogenous METTL3 and NP in SFTSV-infected Huh7 cells using control IgG, anti-METTL3 or anti-NP antibodies, followed by western blotting. (D) Representative confocal microscopy images of mock- or SFTSV-infected HeLa cells (48 hpi, MOI = 1) expressing MYC-tagged NP and FLAG-tagged METTL3 proteins. Nuclei (blue) were strained with DAPI; METTL3 (green) and NP (red) were labeled using anti-FLAG and anti-MYC antibodies, respectively. Scale bar, 10 μm. (E) Nuclear and cytosolic fractions of mock- or SFTSV-infected HEK293T cells (48 hpi, MOI = 1) expressing FLAG-tagged METTL3 were separated and probed by immunoblotting. GAPDH/LAMIN B1 were used as cytoplasmic/nuclear markers, respectively. (F-H) Representative confocal microscopy images of mock- or SFTSV-infected (48 hpi, MOI = 1) HeLa cells expressing MYC-tagged NP and FLAG-tagged YTHDF1/2/3. The nuclei (blue), YTHDF1/2/3 (green), and NP (red) were detected as in panel D. Scale bar, 10 μm. (I-K) Co-IP of SFTSV NP with YTHDF1/2/3 in HEK293T cells co-transfected with FLAG-tagged YTHDF1/2/3 and MYC-tagged NP plasmids for 36 h. Co-IP and western blot were performed as described in panel B. (L-N) Representative confocal microscopy images of mock- or SFTSV-infected HeLa cells expressing MYC-tagged NP and FLAG-tagged IGF2BP1/2/3, detected as in panel D. Scale bar, 10 μm. (O-Q) Co-IP of SFTSV NP with IGF2BP1/2/3 in HEK293T cells co-transfected with FLAG-tagged IGF2BP1/2/3 and MYC-tagged NP plasmids for 36 h. Co-IP and western blot were performed as de [file ppat.1012725.s005.tif]

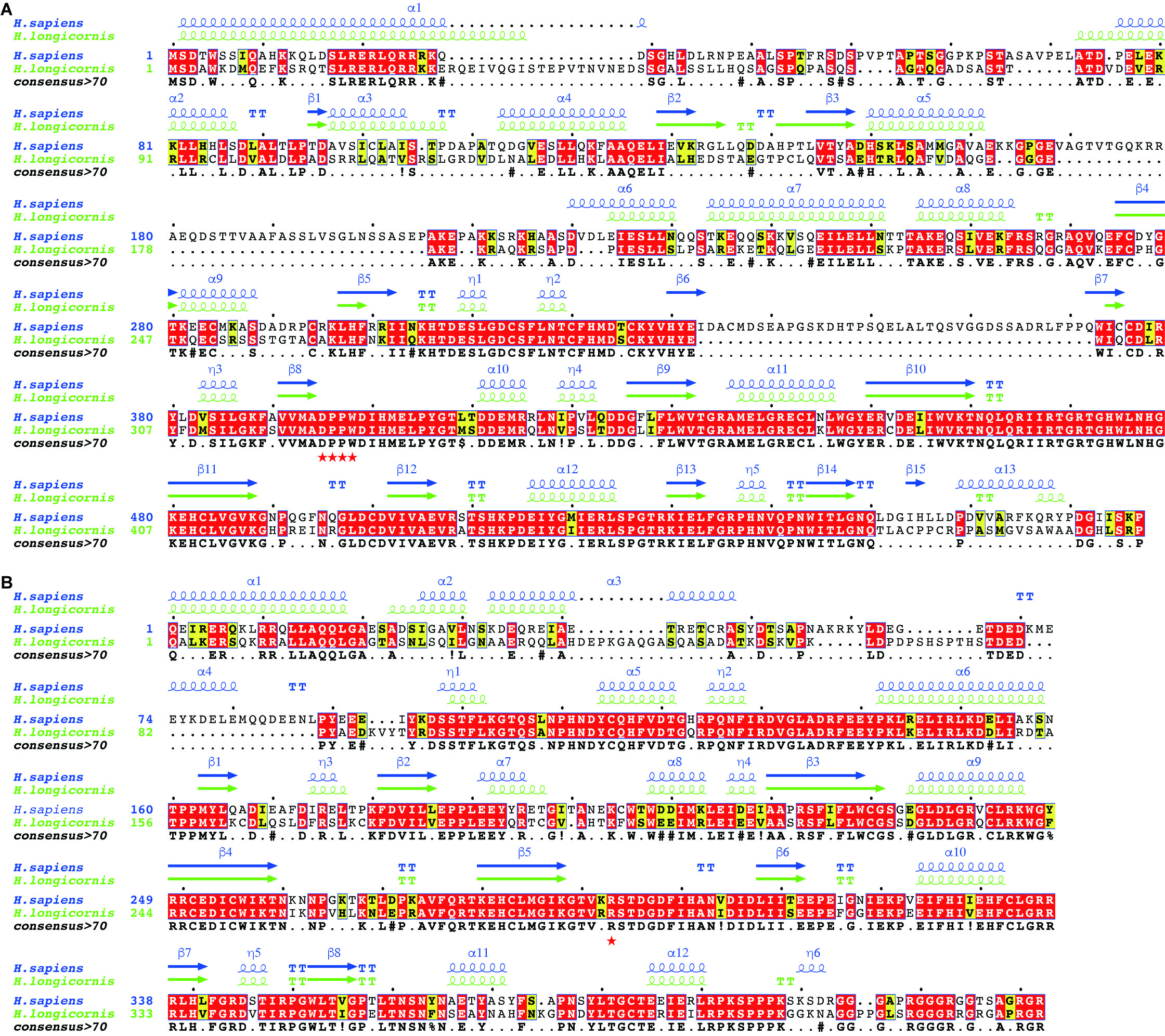

Supplement: S6 Fig — (A) Pairwise sequence alignment between H. Iongicornis HPB48_005528 and H. sapiens METTL3 protein. The DPPW motif is represented by red stars. (B) Pairwise sequence alignment between H. Iongicornis HPB48_008638 and H. sapiens METTL14 protein. The METTL14 R298 residue is represented by a red star. The alignment was performed using the EMBOSS Water web server [119] and visualized by ESPript 3 software [120]. The red highlighted residues are identical, while residues highlighted in yellow are conserved between the two proteins. Secondary structures are represented at the top of the PSA, and the consensus sequence is shown at the bottom. (TIF) [file ppat.1012725.s006.tif]

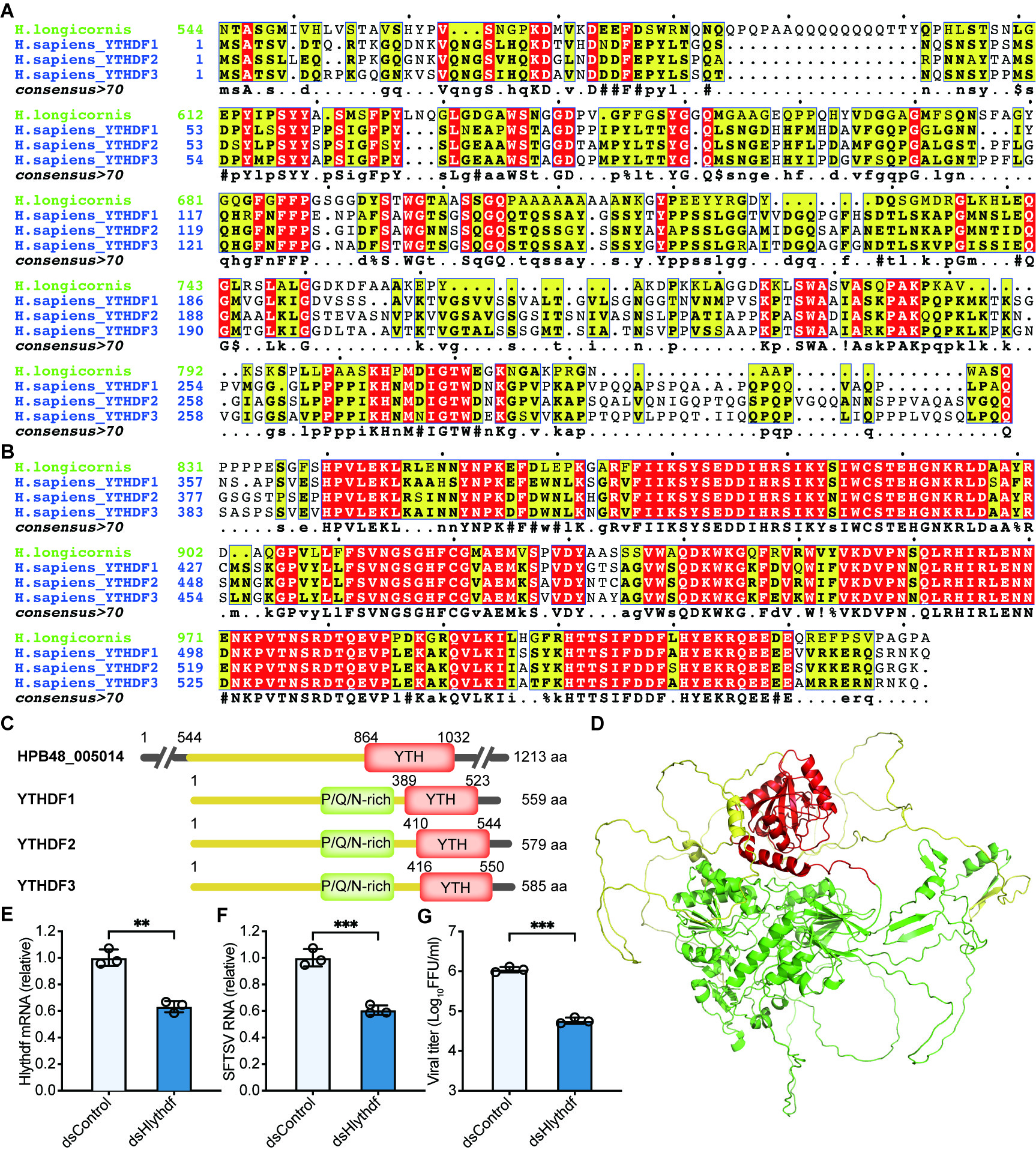

Supplement: S7 Fig — (A-B) Multiple sequence alignment for H. longicornis HPB48_005014 and H. sapiens YTHDF proteins. The alignment was conducted using the Clustal Omega web server [119], and visualized with ESPript 3 software [120]. Identical/conserved residues among the four proteins are highlighted in red/yellow, respectively, with the consensus sequence at the bottom. (A) Alignment of HPB48_005014 at the region in aa 544–829. (B) Alignment of HPB48_005014 at the region in aa 831–1032. (C) Domain architectures of HPB48_005014 and YTHDF proteins. (D) A predicted structure of HPB48_005014 protein generated by ColabFold [72] and visualized using the PyMOL software [71]. Regions in aa 831–1032, aa 544–829, and the remainder are colored with red, yellow, and green, respectively. (E) Efficiency of Hlythdf knockdown. Primary tick cells were transfected with Hlythdf specific (dsHlythdf) or control (dsControl) dsRNA, and relative expressions of Hlythdf mRNA were determined by RT-qPCR with Hlactin as the reference gene. (F-G) The Hlythdf knockdown cells were infected with SFTSV at an MOI of 1. (F) Relative SFTSV RNA levels at 48 hpi were quantified by RT-qPCR with Hlactin as the reference gene. (G) Viral titers in supernatants were assessed by FFA at 48 hpi. Data are representative of three independent experiments and presented as mean ± SD. Statistical significance was determined by student’s t test. **, P < 0.01; ***, P < 0.001. (TIF) [file ppat.1012725.s007.tif]
