# Supplementary material for: N6-methyladenosine RNA modification promotes Severe Fever with Thrombocytopenia Syndrome Virus infection
Source: PLoS Pathog. 2024 Nov 25;20(11):e1012725. doi: 10.1371/journal.ppat.1012725 (PMC11627400; doi:10.1371/journal.ppat.1012725)
Supplement: S1 Table — (DOCX) [file ppat.1012725.s008.docx]

**S1 Table.** Oligonucleotide sequences used for plasmids construction

**Primers used for cloning**

| Construct | Sequence (5'-3') | |
| --- | --- | --- |
| pCAGGS-METTL3-FLAG | F | CATCATTTTGGCAAAGAATTCACCatgtcggacacgtggagc |
|  | R | GTCGTCCTTGTAATCGCTAGCtaaattcttaggtttagagatga |
| pCAGGS-METTL3-∆NTD | F | catcattttggcaaagaattcaccATGtccattgttgaaaaatttcgctctcgag |
|  | R | GTCGTCCTTGTAATCGCTAGCtaaattcttaggtttagagatga |
| pCAGGS-METTL3-∆MTD | F | CATCATTTTGGCAAAGAATTCACCatgtcggacacgtggagc |
|  | R | GTCGTCCTTGTAATCGCTAGCctgtgtaagagcaagctcctgg |
| pCAGGS-METTL3-∆ZFD | F1 | CATCATTTTGGCAAAGAATTCACCatgtcggacacgtggagc |
|  | R1 | CTGGAATCACCTCCGACACTttgttccttggctgttgtag |
|  | F2 | agtgtcggaggtgattcc |
|  | R2 | GTCGTCCTTGTAATCGCTAGCtaaattcttaggtttagagatga |
| pCAGGS-METTL3-MYC | F | CATCATTTTGGCAAAGAATTCACCatgtcggacacgtggagc |
|  | R | aatgagcttttgctcGCTAGCtaaattcttaggtttagagatgataccatctgggt |
| pCAGGS-METTL14-FLAG | F | CATCATTTTGGCAAAGAATTCACCatggatagccgcttgcaggagat |
|  | R | GTCGTCCTTGTAATCGCTAGCtcgaggtggaaagccacctctgt |
| pCAGGS-ALKBH5-FLAG | F | CATCATTTTGGCAAAGAATTCACCatggcggccgccagcggctaca |
|  | R | gtcgtccttgtaatcgctagcgtgccgccgcatcttcacctttcgggcagggctgcctgctgc |
| pCAGGS-FTO-FLAG | F | CATCATTTTGGCAAAGAATTCACCatgaagcgcaccccgact |
|  | R | GTCGTCCTTGTAATCGCTAGCgggttttgcttccagaagct |
| pCAGGS-YTHDF1-FLAG | F | CATCATTTTGGCAAAGAATTCACCatgtcggccaccagcgtg |
|  | R | GTCGTCCTTGTAATCGCTAGCttgtttgtttcgactctgcc |
| pCAGGS-YTHDF2-FLAG | F | CATCATTTTGGCAAAGAATTCACCatgtcggccagcagcctcttg |
|  | R | GTCGTCCTTGTAATCGCTAGCtttcccacgaccttgacgttc |
| pCAGGS-YTHDF3-FLAG | F | CATCATTTTGGCAAAGAATTCACCatgtcagccactagcgtggat |
|  | R | GTCGTCCTTGTAATCGCTAGCttgtttgtttctatttctctccctacgca |
| pCAGGS-IGF2BP1-FLAG | F | CATCATTTTGGCAAAGAATTCACCatgaacaagctttacatcgg |
|  | R | GTCGTCCTTGTAATCGCTAGCcttcctccgtgcctggg |
| pCAGGS-IGF2BP2-FLAG | F | CATCATTTTGGCAAAGAATTCACCatgatgaacaagctttacatcggg |
|  | R | GTCGTCCTTGTAATCGCTAGCcttgctgcgctgtgaggc |
| pCAGGS-IGF2BP3-FLAG | F | CATCATTTTGGCAAAGAATTCACCatgaacaaactgtatatcggaaac |
|  | R | GTCGTCCTTGTAATCGCTAGCcttccgtcttgactgaggtggt |
| pCAGGS-NP-FLAG | F | CATCATTTTGGCAAAGAATTCACCatgtcggagtggtccaggat |
|  | R | GTCGTCCTTGTAATCGCTAGCcaggtttctgtaagcagcagc |
| pCAGGS-NP-MYC | F | catcattttggcaaagaattcaccatgtcggagtggtccaggat |
|  | R | AATGAGCTTTTGCTCGCTAGCcaggtttctgtaagcagcagc |
| pCAGGS-NP-∆NA-MYC | F | CATCATTTTGGCAAAGAATTCACCatgcctgctttgatcatcaagaagc |
|  | R | AATGAGCTTTTGCTCGCTAGCcaggtttctgtaagcagcagc |
| pCAGGS-NP-∆CL-MYC | F | catcattttggcaaagaattcaccatgtcggagtggtccaggat |
|  | R | AATGAGCTTTTGCTCGCTAGCaaggctctgcgcaaccct |
| pCAGGS-NP-∆NL-MYC | F1 | catcattttggcaaagaattcaccatgtcggagtggtccaggat |
|  | R1 | GCAGCACATGTCCAAGTGGGgtcaaggccttcataggc |
|  | F2 | CCCACTTGGACATGTGCT |
|  | R2 | AATGAGCTTTTGCTCGCTAGCcaggtttctgtaagcagcagc |
| pCAGGS-RdRp-FLAG | F | CATCATTTTGGCAAAGAATTCACCatgaacttggaagtgctttgtggt |
|  | R | GTCGTCCTTGTAATCGCTAGCaccccacataatggtgctct |
| dPspCas13b-GGS-ALKBH5 | F | GTGGTGGATCCGGCGGCTCCatggcggccgccagcggct |
|  | R | gtaccgagcccgaattcgtttaaacactaCTTGTCATCGTCGTCCTTGTAATCggatccgtgccgccgcatcttcac |
| pGEX-6P-2-NP(1-106) | F | TTCCAGGGGCCCCTGGGATCCatgtcggagtggtccagg |
|  | R | GTCACGATGCGGCCGCTCGAGcctcactggagtgattgagagc |
| pmirGLO-NP-CDS-WT | F | AGGGCGGCAAGATCGCCGTGatgtcggagtggtccagga |
|  | R | TTGCATGCCTGCAGGTCGACttacaggtttctgtaagcagcagcag |
| pmirGLO-S-genome | F | ccgtgtaattctagttgTTTAAAacacaaagacccccttcatttgga |
|  | R | CTTGCATGCCTGCAGGTCGAacacaaagacccccaaaaaaggaaag |
| pmirGLO-S-antigenome | F | ccgtgtaattctagttgTTTAAAacacaaagacccccaaaaaaggaaag |
|  | R | CTTGCATGCCTGCAGGTCGACacacaaagacccccttcatttgg |
| pXN2-SFTSV-G-WT | F | GAGATCGATCTGTTTACGCGTatgatgaaagtcatctggttctcctct |
|  | R | TCCTCGCCCTTGCTCACCATCTCGAGCGTGATATCTGTTAGTTTTTTTCATACCTAGCAGGATTTGAGctaagccagcttcgtcctcg |
| pAcGFP1-Hlactin-pro-N1 | F | TCAGATCTCGAGCTCAAGCTTggcttcggacgaaggcc |
|  | R | CTCACCATGACCGGTGGATCCgttgactgtttagctgcacagagaag |
| pAcGFP1-Hlmettl3 | F | AGCTAAACAGTCAACGGATCCaccATGGATTACAAGGACGACGATGACAAGtcggacgcttggaaggacat |
|  | R | CCCTTGCTCACCATGACCGGttgacgtggcacgggggt |
| pAcGFP1-Hlmettl14 | F | AGCTAAACAGTCAACGGATCCaccatgGATTACAAGGACGACGATGACAAGagcggggaactcacggtcag |
|  | R | CCCTTGCTCACCATGACCGGgcgtcctctcggggctcccct |
| pAcGFP1-Ythdf | F | AGCTAAACAGTCAACGGATCCaccatgGATTACAAGGACGACGATGACAAGctttcctctcgctgttccagt |
|  | R | CCCTTGCTCACCATGACCGGaagaacgaaagaaacgtcccct |

**Primers used for mutagenesis**

| Construct | Sequence (5'-3') | |
| --- | --- | --- |
| pCAGGS-METTL3-APPA | F | GCAGTTGTGATGGCTGccccacccgcGGATATTCACATGGAA |
|  | R | TTCCATGTGAATATCCgcgggtggggCAGCCATCACAACTGC |
| pCAGGS-ALKBH5-H204A | F | GGGTCCACggcAGACACGATGCAGCCGC |
|  | R | GCGGCTGCATCGTGTCTgccGTGGACCC |
| pCAGGS-NP-C69T | F | cagtcaaattgagctgctgagcttgaggaTttcgcgagag |
|  | R | ctctcgcgaaAtcctcaagctcagcagctcaatttgactg |
| pCAGGS-NP-A273T | F | ggttgatggcactccaagagaaatatggTctggttgagagg |
|  | R | cctctcaaccagAccatatttctcttggagtgccatcaacc |
| pcDNA3.1(+)-T7-S-vRNA- | F | cttgggttacctgagatcctaataactggTctatccccaattc |
| A556T | R | gaattggggatagAccagttattaggatctcaggtaacccaag |
| pcDNA3.1(+)-T7-S-vRNA- | F | gcgtaagcctctattggaTtgttgggaTttctttattcctgtccgc |
| C673T+C682T | R | gcggacaggaataaagaaAtcccaacaAtccaatagaggcttacgc |
| pcDNA3.1(+)-T7-S-vRNA- | F | ctcgagtcagggcaaagacaatgatgaaTtttgtatccttcaccc |
| C1538T | R | gggtgaaggatacaaaAttcatcattgtctttgccctgactcgag |
| pXN2-SFTSV-G-T496G | F | aaagctgaggctgacaatgtagctatggacGccctccaaga |
|  | R | tcttggagggCgtccatagctacattgtcagcctcagcttt |
| pXN2-SFTSV-G-A1390T | F | gttggatgtgaaatgaggggggcacccagaTtggcagtctc |
|  | R | gagactgccaAtctgggtgcccccctcatttcacatccaac |
| pXN2-SFTSV-G-A1945T | F | atccccagaccccaattgccttaaggacGttggtgagtaac |
|  | R | gttactcaccaaCgtccttaaggcaattggggtctggggat |

**Primers used for shRNA constructs**

| shRNA | Sequence (5'-3') | |
| --- | --- | --- |
| shMETTL3-1 | F | gatccGCTACAGATCCTGAGTTAGTTCAAGAGACTAACTCAGGATCTGTAGCTTTTTTg |
|  | R | aattcAAAAAAGCTACAGATCCTGAGTTAGTCTCTTGAACTAACTCAGGATCTGTAGCg |
| shMETTL3-2 | F | gatccGAGCCAGCCAAGAAATCAATTCAAGAGATTGATTTCTTGGCTGGCTCTTTTTTg |
|  | R | aattcAAAAAAGAGCCAGCCAAGAAATCAATCTCTTGAATTGATTTCTTGGCTGGCTCg |
| shMETTL14-1 | F | gatccGGATGAAGGAGAGACAGATTTCAAGAGAATCTGTCTCTCCTTCATCCTTTTTTg |
|  | R | aattcAAAAAAGGATGAAGGAGAGACAGATTCTCTTGAAATCTGTCTCTCCTTCATCCg |
| shMETTL14-2 | F | gatccGCTGGACTTGGGATGATATTTCAAGAGAATATCATCCCAAGTCCAGCTTTTTTg |
|  | R | aattcAAAAAAGCTGGACTTGGGATGATATTCTCTTGAAATATCATCCCAAGTCCAGCg |
| shALKBH5-1 | F | gatccGCGCAACAAGTACTTCTTCTTCAAGAGAGAAGAAGTACTTGTTGCGCTTTTTTg |
|  | R | aattcAAAAAAGCGCAACAAGTACTTCTTCTCTCTTGAAGAAGAAGTACTTGTTGCGCg |
| shALKBH5-2 | F | gatccGTGAGAAGCTCAAGTCCATTTCAAGAGAATGGACTTGAGCTTCTCACTTTTTTg |
|  | R | aattcAAAAAAGTGAGAAGCTCAAGTCCATTCTCTTGAAATGGACTTGAGCTTCTCACg |
| shFTO-1 | F | gatccGCTCGCATCCTCATTGGTAATTCAAGAGATTACCAATGAGGATGCGAGTTTTTTg |
|  | R | aattcAAAAAACTCGCATCCTCATTGGTAATCTCTTGAATTACCAATGAGGATGCGAGCg |
| shFTO-2 | F | gatccAACACACCGAGGCTGAAATTTCAAGAGAATTTCAGCCTCGGTGTGTTTTTTTTg |
|  | R | aattcAAAAAAAACACACCGAGGCTGAAATTCTCTTGAAATTTCAGCCTCGGTGTGTTg |
| shYTHDF1-1 | F | gatccGAGTAACAGTTACCCCTCATTCAAGAGATGAGGGGTAACTGTTACTCTTTTTTg |
|  | R | aattcAAAAAAGAGTAACAGTTACCCCTCATCTCTTGAATGAGGGGTAACTGTTACTCg |
| shYTHDF1-2 | F | gatccGGGGAACAACATCTATCAGTTCAAGAGACTGATAGATGTTGTTCCCCTTTTTTg |
|  | R | aattcAAAAAAGGGGAACAACATCTATCAGTCTCTTGAACTGATAGATGTTGTTCCCCg |
| shYTHDF2-1 | F | gatccGCTGCCATGTCAGATTCCTATTCAAGAGATAGGAATCTGACATGGCAGTTTTTTg |
|  | R | aattcAAAAAACTGCCATGTCAGATTCCTATCTCTTGAATAGGAATCTGACATGGCAGCg |
| shYTHDF2-2 | F | gatccGGCCCAATAATGCATATACTTCAAGAGAGTATATGCATTATTGGGCCTTTTTTg |
|  | R | aattcAAAAAAGGCCCAATAATGCATATACTCTCTTGAAGTATATGCATTATTGGGCCg |
| shYTHDF3-1 | F | gatccGCCATACTTAAGTAGCCAGTTCAAGAGACTGGCTACTTAAGTATGGCTTTTTTg |
|  | R | aattcAAAAAAGCCATACTTAAGTAGCCAGTCTCTTGAACTGGCTACTTAAGTATGGCg |
| shYTHDF3-2 | F | gatccAGACCAGCCTATGCCATATTTCAAGAGAATATGGCATAGGCTGGTCTTTTTTTg |
|  | R | aattcAAAAAAAGACCAGCCTATGCCATATTCTCTTGAAATATGGCATAGGCTGGTCTg |

**Primers used for sgRNA constructs**

| sgRNA | Sequence (5'-3') | |
| --- | --- | --- |
| S_vRNA g1 | F | caccGCCAACAGTCCAATAGAGGCTTAC |
|  | R | caacGTAAGCCTCTATTGGACTGTTGGC |
| S_vRNA g2 | F | caccGTAGTCCAGTTATTAGGATCTCAG |
|  | R | caacCTGAGATCCTAATAACTGGACTAC |
| S_vRNA g3 | F | caccGTCCAACAACTGCTCAAGTCCGG |
|  | R | caacCCGGACTTGAGCAGTTGTTGGAC |
| S_vRNA g4 | F | caccGCATCAAGAAGCTGAAGGAGACAG |
|  | R | caacCTGTCTCCTTCAGCTTCTTGATGC |
| M_vRNA g1 | F | caccGATAGCTACATTGTCAGCCTCAGC |
|  | R | caacGCTGAGGCTGACAATGTAGCTATC |
| M_vRNA g2 | F | caccGCCCCTCATTTCACATCCAACTCC |
|  | R | caacGGAGTTGGATGTGAAATGAGGGGC |
| M_vRNA g3 | F | caccGTCATATTTTGTTCCTGATGCCCG |
|  | R | caacCGGGCATCAGGAACAAAATATGAC |
| M_vRNA g4 | F | caccGATGTCTGCACTTCACAGCACCTG |
|  | R | caacCAGGTGCTGTGAAGTGCAGACATC |
| M_vRNA g5 | F | caccGAATGTCCTTAAGGCAATTGGGGT |
|  | R | caacACCCCAATTGCCTTAAGGACATTC |
| M_vRNA g6 | F | caccGGAAGTCTAGGACTGAGTCAGGA |
|  | R | caacTCCTGACTCAGTCCTAGACTTCC |
| M_vRNA g7 | F | caccGATATGATAGTTCCTGGGCCTTCA |
|  | R | caacTGAAGGCCCAGGAACTATCATATC |
| L_vRNA g1 | F | caccGTCAGACATGGATGAAGTCCGATC |
|  | R | caacGATCGGACTTCATCCATGTCTGAC |
| L_vRNA g2 | F | caccGTATGTCATTATCAAGCCGACCAA |
|  | R | caacTTGGTCGGCTTGATAATGACATAC |
| METTL3_gRNA1 | F | caccGTCTGAACCAACAGTCCACTA |
|  | R | aaacTAGTGGACTGTTGGTTCAGAC |
| METTL3_gRNA2 | F | caccGTCAGCATAGGTTACAAGAGT |
|  | R | aaacACTCTTGTAACCTATGCTGAC |
