# Supplementary material for: N6-methyladenosine RNA modification promotes Severe Fever with Thrombocytopenia Syndrome Virus infection
Source: PLoS Pathog. 2024 Nov 25;20(11):e1012725. doi: 10.1371/journal.ppat.1012725 (PMC11627400; doi:10.1371/journal.ppat.1012725)
Supplement: S2 Table — (DOCX) [file ppat.1012725.s009.docx]

**S2 Table.** Primers for preparation of DNA Templates for In Vitro Transcription.docx

| Primers | Sequence (5'-3') |
| --- | --- |
| S-vRNA-T7F | acacaaagaacccccaaaaaaggaaag |
| S-vRNA-T7R | TAATACGACTCACTATAGGGacacaaagacccccttcatttggaaacc |
| S-cRNA-T7F | TAATACGACTCACTATAGGGacacaaagaacccccaaaaaaggaaag |
| S-cRNA-T7R | acacaaagacccccttcatttggaaacc |
| Control-dsRNA-T7F | TAATACGACTCACTATAGGGatgggagtcaaagttctgtt |
| Control-dsRNA-T7R | TAATACGACTCACTATAGGGttagtcaccaccggccccct |
| Hlmettl3-dsRNA-T7F | TAATACGACTCACTATAGGGtcggacgcttggaaggacat |
| Hlmettl3-dsRNA-T7R | TAATACGACTCACTATAGGGgcgacagcagcgactcgatc |
| Hlmettl14-dsRNA-T7F | TAATACGACTCACTATAGGGgtgcggatgcgaccaaagatt |
| Hlmettl14-dsRNA-T7R | TAATACGACTCACTATAGGGtcttgatgttggtcttgatcc |
| Hlythdf-dsRNA-T7F | TAATACGACTCACTATAGGGgcagcggaagtaccagagaat |
| Hlythdf-dsRNA-T7R | TAATACGACTCACTATAGGGaggtaaggaaaggacatggac |
