# Supplementary material for: N6-methyladenosine RNA modification promotes Severe Fever with Thrombocytopenia Syndrome Virus infection
Source: PLoS Pathog. 2024 Nov 25;20(11):e1012725. doi: 10.1371/journal.ppat.1012725 (PMC11627400; doi:10.1371/journal.ppat.1012725)
Supplement: S3 Table — (DOCX) [file ppat.1012725.s010.docx]

**S3 Table.** Primer sets for strand-specific reverse transcription qPCR.docx

| Primers | Purpose | Sequence (5'-3') | Genome position (Genomic sense) |
| --- | --- | --- | --- |
| S-vRNA-tag-RT | RT | GGCCGTCATGGTGGCGAATcgcaaaggagtgatcatgtcg | 28-48 |
| vRNA-tag | qPCR | GGCCGTCATGGTGGCGAAT |  |
| S-vRNA-R | qPCR | aactttgtatccttcacccaa | 189-209 |
| S-cRNA-tag-RT | RT | GCTAGCTTCAGCTAGGCATCccccttcatttggaaaccatg | 1714-1734 |
| cRNA-tag | qPCR | GCTAGCTTCAGCTAGGCATC |  |
| S-cRNA-R | qPCR | aagagcactcaacgaggt | 1593-1610 |
| M-vRNA-tag-RT | RT | GGCCGTCATGGTGGCGAATcgcaaaggagtgatcatgtcg | 135-153 |
| vRNA-tag | qPCR | GGCCGTCATGGTGGCGAAT |  |
| M-vRNA-R | qPCR | cccaacgtagccttgaaattgc | 213-234 |
| M-cRNA-tag-RT | RT | GCTAGCTTCAGCTAGGCATCccccttcatttggaaaccatg | 3359-3377 |
| cRNA-tag | qPCR | GCTAGCTTCAGCTAGGCATC |  |
| M-cRNA-R | qPCR | atgggcaaatttctggtctagtga | 3241-3264 |
| L-vRNA-tag-RT | RT | GGCCGTCATGGTGGCGAATcgcaaaggagtgatcatgtcg | 8-28 |
| vRNA-tag | qPCR | GGCCGTCATGGTGGCGAAT |  |
| L-vRNA-R | qPCR | tgttacaccagtggcatcgac | 134-154 |
| L-cRNA-tag-RT | RT | GCTAGCTTCAGCTAGGCATCccccttcatttggaaaccatg | 6341-6359 |
| cRNA-tag | qPCR | GCTAGCTTCAGCTAGGCATC |  |
| L-cRNA-R | qPCR | atcagttctgcgactcca | 6216-6233 |

Tagged RT primers were used for strand-specific reverse transcription. The Tag sequence is from Brennan et al., 2015 [63] and is in capital letters and underlined.
