# Supplementary material for: N6-methyladenosine RNA modification promotes Severe Fever with Thrombocytopenia Syndrome Virus infection
Source: PLoS Pathog. 2024 Nov 25;20(11):e1012725. doi: 10.1371/journal.ppat.1012725 (PMC11627400; doi:10.1371/journal.ppat.1012725)
Supplement: S4 Table — (DOCX) [file ppat.1012725.s011.docx]

**S4 Table.** Oligonucleotide sequences of primers used in MeRIP-qPCR

| Primers | Sequence (5'-3') | Genome position (Genomic sense) |
| --- | --- | --- |
| S-vRNA-m6A-F | GCCCTCCTCCAGATAGAGTCA | 1147-1167 |
| S-vRNA-m6A-RT-R | AAAGAAGTCCCAACAGTCCAA | 1057-1077 |
| M-vRNA-m6A-F | GGAGTTGGATGTGAAATGAGGGG | 2000-2022 |
| M-vRNA-m6A-RT-R | TCATATTTTGTTCCTGATGCCCG | 1918-1940 |
| L-vRNA-m6A-F | TCCAAGAAAGGGCTCAATGTC | 1360-1380 |
| L-vRNA-m6A-RT-R | ATAACCTATAGGCGATTCAA | 1232-1251 |
| S-cRNA-m6A-F | GAAGGAGACAGGTGGAGA | 168-185 |
| S-cRNA-m6A-RT-R | TCTTGGAGTGCCATCAAC | 288-305 |
| M-cRNA-m6A-F | ATAGCTACATTGTCAGCCTCAGC | 2888-2910 |
| M-cRNA-m6A-RT-R | ATGTCCCCCACAGTTCAGCTGGC | 2939-2961 |
| L-cRNA-m6A-F | GACGCCCAGATGAACTTGGAA | 8-28 |
| L-cRNA-m6A-RT-R | TGTTACACCAGTGGCATCGAC | 134-154 |
| HPRT1-F | CATTATGCTGAGGATTTGGAAAGG |  |
| HPRT1-R | CTTGAGCACACAGAGGGCTACA |  |

The reverse transcription primers are also used as reverse primers for quantitative qPCR.
