# Supplementary material for: N6-methyladenosine RNA modification promotes Severe Fever with Thrombocytopenia Syndrome Virus infection
Source: PLoS Pathog. 2024 Nov 25;20(11):e1012725. doi: 10.1371/journal.ppat.1012725 (PMC11627400; doi:10.1371/journal.ppat.1012725)
Supplement: S5 Table — (DOCX) [file ppat.1012725.s012.docx]

**S5 Table.** Oligonucleotide sequences of primers for RT-qPCR

| Gene | Sequence (5'-3') | |
| --- | --- | --- |
| ACTB | F | CACCATTGGCAATGAGCGGTTC |
|  | R | AGGTCTTTGCGGATGTCCACGT |
| METTL3 | F | CTATCTCCTGGCACTCGCAAGA |
|  | R | GCTTGAACCGTGCAACCACATC |
| METTL14 | F | CTGAAAGTGCCGACAGCATTGG |
|  | R | CTCTCCTTCATCCAGATACTTACG |
| ALKBH5 | F | CCAGCTATGCTTCAGATCGCCT |
|  | R | GGTTCTCTTCCTTGTCCATCTCC |
| FTO | F | CCAGAACCTGAGGAGAGAATGG |
|  | R | CGATGTCTGTGAGGTCAAACGG |
| YTHDF1 | F | CAAGCACACAACCTCCATCTTCG |
|  | R | GTAAGAAACTGGTTCGCCCTCAT |
| YTHDF2 | F | TAGCCAGCTACAAGCACACCAC |
|  | R | CAACCGTTGCTGCAGTCTGTGT |
| YTHDF3 | F | GCTACTTTCAAGCATACCACCTC |
|  | R | ACAGGACATCTTCATACGGTTATTG |
| SFTSV S | F | CAATTTGACTGAGCTTGAGGACTTCGC |
|  | R | ACATTTTCCCTGATGCCTTGACGA |
| SFTSV NP | F | GAAGGAGACAGGTGGAGA |
|  | R | TCTTGGAGTGCCATCAAC |
| SFTSV G | F | ccacctgcttgggtactct |
|  | R | cccaacgtagccttgaaattgc |
| HPRT1 | F | CATTATGCTGAGGATTTGGAAAGG |
|  | R | CTTGAGCACACAGAGGGCTACA |
| R-luciferase | F | GTCTGGCCTTTCACTACTCCT |
|  | R | CTTGATCAGGGCGATATCCTC |
| F-luciferase | F | TACCTACGCCGAGTACTTCGAGA |
|  | R | CACCGATGAACAGGGCACCCAA |
| Hlactin | F | CAGGGAGTCATGGTTGGCAT |
|  | R | CATGTCGTCCCAGTTGGTCA |
| Hlmettl3 | F | GCGACTGCTCCTTCCTCAACACC |
|  | R | GCCATCACCACCGAGAACTTGCC |
| Hlmettl14 | F | CCGTGTTCCAGCGCACCA |
|  | R | AAGATCTCCTCAGGCTTCTCG |
| Hlythdf | F | TACAACCCCAAGGAGTTTGACC |
|  | R | CTCCGTCGAGCACCAGA |
