# Supplementary material for: N6-methyladenosine RNA modification promotes Severe Fever with Thrombocytopenia Syndrome Virus infection
Source: PLoS Pathog. 2024 Nov 25;20(11):e1012725. doi: 10.1371/journal.ppat.1012725 (PMC11627400; doi:10.1371/journal.ppat.1012725)
Supplement: Attachment — Submitted filename: Response letter to reviewers PP-D-24-00959 (1).docx [file ppat.1012725.s013.docx]

September 20, 2024

Editors

*PLOS Pathogens*
RE: PPATHOGENS-D-24-00959

Dear Editors,

Thank you for the comments on our manuscript entitled “**N6-methyladenosine RNA modification promotes Severe Fever with Thrombocytopenia Syndrome Virus infection**”(No. PPATHOGENS-D-24-00959). We are thankful to all the editors and reviewers for their constructive criticisms. We have heeded their specific comments, and conducted all the suggested experiments to address the questions. Our point-by-point responses to the editor and reviewers’ criticisms were provided below and all the specific changes have been highlighted in the revised manuscript text.

**Part I – Summary**

Reviewer #1: Severe Fever with Thrombocytopenia Syndrome Virus (SFTSV), or Dabie bandavirus (DBV), is a tick-transmitted (-) ssRNA virus that causes severe fever with thrombocytopenia syndrome with a mortality rate up to 12%. N6-methyladenosine (m6A) is a prevalent epigenetic modification of cellular and viral RNA, regulating gene expression. However, the role of m6A modification during SFTSV infection remains elusive. In this manuscript, Chen et al. discovered the m6A modification to SFTSV genome, which facilitates viral replication. Approximately 0.32% of A bases in SFTSV RNA were m6A methylated, similar to the level in host mRNA. siRNA-mediated knockdown oh m6A writer, METTL3 or METTL14, reduced, while overexpression promoted viral replication in multiple cell lines. Knockdown of a m6A eraser, ALKBH5, increased, while overexpression reduced viral replication. iTRAQ-based quantitative proteomic analysis revealed that SFTSV infection upregulated these m6A writers and erasers. Mechanistically, SFTSV nucleoprotein (NP) interacted with METTL3, and m6A modification of viral RNA genome enhanced viral RNA stability and translation efficiency. The authors identified the m6A targets in the genome of SFTSV and made mutant viruses (VSV-pseudotyped). The mutant viruses were less infective and pathogenic in mice and cell cultures, when compared to their parental strain. Interestingly, the m6A modification to SFTSV genome, and its role in viral replication were recapitulated in tick cells. This is a very comprehensive study with rigorous approaches and analyses, novel and solid results, reasonable conclusions. The manuscript is well organized and clearly written.

Reviewer #2: The tick-borne Thrombocytopenia Syndrome Virus (SFTSV) can cause sever fever and serious health concerns to human health. This manuscript used multiple research techniques to identify that N6-methyladenosine (m6A) RNA modification promotes SFTSV infection in human cells, mice, and ticks. The authors provided several lines of evidence and elegantly supported their findings. As the reviewers, I only have some minor concerns with the style of writing and request a minor revision.

Reviewer #3: N6-methyladenosine (m6A) is a very common post-transcriptional modification found in both host and viral RNAs. It has been shown to regulate various viral infections in addition to the roles in modulation of cellular biological processes. Previous studies have reported m6A modification in SFTSV genomic RNAs and shown the potential involvement of multiple related regulatory proteins during the SFTSV infection. However, detailed functional implications need further depiction. In this paper by Chen et al., several possible proviral roles of m6A modification in NP/S and GP/M RNAs are proposed. Additionally, positive regulation of the viral infection potentially by m6A is also implicated in H. longicornis tick cells. The manuscript is interesting and clearly written on the whole. However, there are several significant concerns (especially regarding the inconsistency of some major results and lacking of mechanism elucidation as well as an appropriate in vivo evaluation) that require to be comprehensively addressed.

We are grateful to all reviewers for their valuable comments on our manuscript, and we will answer each of the specific questions below.

**Part II – Major Issues: Key Experiments Required for Acceptance**

**1. Reviewer #1: The authors have presented comprehensive and convincing results to support their conclusions.**

**2. Reviewer #2: NA**

**3. Reviewer #3:**

**1)** SFTSV is a negative-sense “single-stranded” RNA virus, which means that the virions contain negative-sense genomic RNA segments. How are m6A modifications in antigenomic RNAs abundantly detected and quantified in the virion particles? If that is the case, further investigations and additional data and discussions are required here.

Moreover, I suggest integrating the results of intracellular m6A methylome sequencing analysis into the main text rather than placing them in supplementary materials. It is unclear whether packaging integration of antisense RNA can occur (previous evidence does not support it for SFTSV), what the efficiency is, and whether it is uniform. Additionally, please carefully compare, analyze, and discuss the similarities and differences between the sequencing results in this article and those in the previous paper, and provide corresponding explanations.

Thank you for your comments. Our MeRIP-seq results show that small amounts of +ssRNA do exist inside the viral particles, although the amount of +ssRNA is much lower compared to -ssRNA (S1A-S1B Fig).

1) We ruled out the possibility of cellular RNA contamination because we could not detect any cellular RNAs such as GAPDH in the virion RNA samples (S1C Fig).

2) As reported, +ssRNA was also detected in virion of Rift Valley fever virus, another Bunyavirus [1, 2].

3) In several other MeRIP-seq studies, +ssRNA was also detected in the virions of, other -ssRNA viruses, such as Respiratory syncytial virus (RSV), Human metapneumovirus (HMPV), and VSV [3-5]. Based on these evidence, we believed our MeRIP-seq results for SFTSV virions are interpretable.

But, we agreed with the reviewer’s comments, this is not the key point that we're trying to address here. We just want to emphersize that the SFTSV genomic RNAs in virions are m^6^A modified. So we moved the data of +ssRNA methylation found in virions into the supplementary data (new S1D-S1F Fig). We also integrated the results of intracellular m^6^A methylome sequencing into the New Figure 1D-1F as you suggested and provided further analysis and corresponding explanations in the relevent section (lines 168-196 and lines 720-734).

New Fig 1D-1F

**2)** Fig 2 illustrates the positive role of m6A modification in SFTSV infection, which is a major result of the paper. However, the data presented here are not sufficiently reliable or comprehensive.

For instance, only one shRNA was used in the knockdown experiment for functional validation, further verification of the actual knockdown effect by WB is lacking, and many results show a very weak impact (especially in the quantification of RNA copies, difference of only ~0.1 lg was observed in several comparisons), among other issues.

Additionally, even under the same experimental conditions within the same cells, if we only look at the control groups, the quantitative differences in RNA copy numbers are extremely large and severely inconsistent. For example, in Figures 2A and 2C, the shCtrl groups show lg RNA copies: in Huh7 cells, 6.7 vs 5.2; in HeLa cells, 3.5 VS 5; in HEK293T, 3.5 VS 4.8. Furthermore, in Figures 2B and 2D, the Vector groups demonstrate: in Huh7 cells, 4.5 lg vs 6 lg; in HeLa cells, 3.9 lg VS 6.9 lg (meaning 1000-fold difference in virus yields under the same experimental settings); in HEK293T, 3.5 lg VS 5.2 lg, etc.

The results in Fig 2H and 2I ruling out the involvement of the interferon system are also a bit weak. Why were cells with a heterozygous STAT1 deficiency (STAT1+/-) used, instead of cells with a homozygous STAT1 defect (STAT1-/-)?

These severely affect the quality and soundness of the paper and may need to be thoroughly checked and reinforced.

Thank you for your concerns regarding the reliability and comprehensiveness of the data presented in Fig 2, and we will address these concerns with additional clarifications below.

**2.1)** For instance, only one shRNA was used in the knockdown experiment for functional validation, further verification of the actual knockdown effect by WB is lacking, and many results show a very weak impact (especially in the quantification of RNA copies, difference of only ~0.1 lg was observed in several comparisons), among other issues.

Thank you for your comments regarding the shRNA knockdown experiment. In fact, in Supplementary S2 Fig, we employed two distinct shRNAs targeting each m^6^A regulators, and we validated their knockdown efficiency using both RT-qPCR and western blot. We selected the shRNA with the higher knockdown efficiency for subsequent functional validation experiments. While some comparisons showed relatively small magnitude, these differences were statistically significant. Additionally, to further confirm the role of m^6^A in SFTSV infection, as you suggested, we have performed functional validation experiments using METTL3 knockout cells (see below, new Fig 2F-2I). These experiments demonstrated a more pronounced effect on SFTSV replication. And we believe these differences have biological relevance in the context of SFTSV infection, consistent with previous studies on the role of m^6^A in regulating replication of RNA viruses such as EV71, RSV and HMPV [3, 4, 6].

**2.2)** Additionally, even under the same experimental conditions within the same cells, if we only look at the control groups, the quantitative differences in RNA copy numbers are extremely large and severely inconsistent. For example, in Figures 2A and 2C, the shCtrl groups show lg RNA copies: in Huh7 cells, 6.7 vs 5.2; in HeLa cells, 3.5 VS 5; in HEK293T, 3.5 VS 4.8. Furthermore, in Figures 2B and 2D, the Vector groups demonstrate: in Huh7 cells, 4.5 lg vs 6 lg; in HeLa cells, 3.9 lg VS 6.9 lg (meaning 1000-fold difference in virus yields under the same experimental settings); in HEK293T, 3.5 lg VS 5.2 lg, etc.

Thanks for pointing out this issue. The reason why the viral RNA copy numbers in the supernatants are not exactly consistent may relate to the following facts: 1) Different cell lines and different virus batches were used. 2) The experiments presented in different panels were not conducted simultaneously, which could introduce batch effects in viral replication kinetics. 3) The CT values will be different when the baselines for the qPCR are taken differently.

Although the raw RNA copy numbers in the control groups show variability, it is important to emphasize that our analysis focuses on **relative comparisons** between the experimental groups and their respective controls within each cell line and condition. This method of comparison remains valid, and does not affect the internal consistency of the results.

In order to avoid potential confusions here, we standardized the data by setting the mean value of the Control group to 1 for each group and show the relative copy of viral RNAs isolated from equal volumes of the supernatants (new Fig 2A-2D). This allows us to present the relative viral RNA levels across experimental groups, thus eliminating potential biases introduced by variability in absolute RNA copy numbers.

New Fig 2A-2D

For better supporting our conclusion here, we also generated METTL3^-/-^ cells, and showed the phenotypes of SFTSV replication in these cells (lines 257-262). These results were included in the new revised manuscript as New Fig 2F-2I.

New Fig 2F-2I

**2.3)** The results in Fig 2H and 2I ruling out the involvement of the interferon system are also a bit weak. Why were cells with a heterozygous STAT1 deficiency (STAT1^+/-^) used, instead of cells with a homozygous STAT1 defect (STAT1^-/-)^?

These severely affect the quality and soundness of the paper and may need to be thoroughly checked and reinforced.

Thank you for your insightful comment. We have now repeated the experiments using STAT1^-/-^ cells to directly test the effect of METTL3 overexpression in a more complete absence of the interferon signaling pathway.

Our new results show that overexpression of METTL3 in STAT1^-/-^ cells similarly promotes SFTSV replication. We have included these new results in the revised manuscript (new Fig 2K).

New Fig 2K

**3)** Figure 4 shows a potential interaction between A and B, which is considered a mechanism analysis. However, the data presented here are also insufficient and uncertain at the current state.
 For instance, both METTL3 and NP are RNA-binding proteins. Is the interaction between METTL3 and NP mediated by RNA in a non-specific manner? Endogenous METTL3 interaction/colocalization with NP also need to be tested in the context of SFTSV infection. Also, the biological significance of this interaction still requires further experimental validation. Does METTL3 interact with other viral proteins? That is, at least, a control with other viral proteins is required. Further, in Figure 4H, the authors claim that the ZFD domain is crucial for the interaction between NP and METTL3, but the precipitation of the delZFD (when used as bait protein) itself is significantly lower than other truncations or the wild-type protein. This result needs to be repeated and verified. Moreover, from the results in Figure 4K, it can be seen that even such a large deletion of the whole MTD domain (which totally deprives of methyltransferase activity) still retains the pro-viral activity. This activity, however, is only slightly lower than that of the full-length protein, despite its expression being significantly less. These suggest that even with a complete loss of the enzymatic activity, there is no significant reduction in its pro-viral function. Thus, the extent to which the observed pro-viral activity is related to its methyltransferase activity remains uncertain.

Thank you for your concerns regarding the the data presented in Fig 4, and we will address these concerns below.

**3.1)** For instance, both METTL3 and NP are RNA-binding proteins. Is the interaction between METTL3 and NP mediated by RNA in a non-specific manner?

Thank you for raising this important point. To address whether the interaction between METTL3 and NP is mediated by RNA in a non-specific manner, we conducted an RNase treatment followed by co-immunoprecipitation (Co-IP). Our results demonstrated that METTL3 still interacts with NP after RNase treatment, indicating that this interaction is RNA-independent. We have clarified this point in the revised manuscript (lines 336-340) and put this result in new Fig 4E.

New Fig 4E

**3.2)** Endogenous METTL3 interaction/colocalization with NP also need to be tested in the context of SFTSV infection.

Thank you for your valuable feedback. As suggested, we have performed co-localization studies using immunofluorescence microscopy to visualize the interaction between endogenous METTL3 and NP in SFTSV-infected cells. We have highlighted this point in the revised manuscript (lines 330-333) and put this result into the new Fig 4C.

New Fig 4C

**3.3)** Also, the biological significance of this interaction still requires further experimental validation. Does METTL3 interact with other viral proteins? That is, at least, a control with other viral proteins is required.

Thank you for your valuable suggestion. In response to your comment, we performed additional Co-IP experiments to assess the interaction between METTL3 and other viral proteins, including NP, NSs, and G proteins. Our results, as shown in Supplementary Figure S5B, demonstrated that METTL3 interacts not only with NP, but also with G protein. Notably, the interaction between METTL3 and NP is comparable in strength to that of METTL3 with G. And it seems that METTL3 may not interact with NSs.

These new findings further support the involvement of METTL3 in the viral life cycle through interactions with multiple SFTSV proteins, providing a broader context for its role. We have updated the manuscript accordingly (lines 327-330) and included these new data in new S5B Fig. We hope this additional validation addresses your concern.

New S5B Fig

**3.4)** Further, in Figure 4H, the authors claim that the ZFD domain is crucial for the interaction between NP and METTL3, but the precipitation of the delZFD (when used as bait protein) itself is significantly lower than other truncations or the wild-type protein. This result needs to be repeated and verified.

We appreciate the reviewer’s thoughtful critique and for highlighting the need to verify the results shown in Figure 4H. In response to the concern, we have repeated the experiment to confirm the findings. The results from the repeated experiments are consistent with our initial findings. We have updated the corresponding panel in the new Fig 4G.

New Fig 4G

**3.5)** Moreover, from the results in Figure 4K, it can be seen that even such a large deletion of the whole MTD domain (which totally deprives of methyltransferase activity) still retains the pro-viral activity. This activity, however, is only slightly lower than that of the full-length protein, despite its expression being significantly less. These suggest that even with a complete loss of the enzymatic activity, there is no significant reduction in its pro-viral function. Thus, the extent to which the observed pro-viral activity is related to its methyltransferase activity remains uncertain.

Thank you for raising this important point. We have carefully repeated these experiments. Although the differences between Vec Vs ΔMTD (methyltransferase activity defective one) are smaller than before (new Fig 4J), the ΔMTD group still have a minor pro-viral effect on SFTSV infection. After checking with literatures, we found that METTL3 may also contribute to mRNA translation in an methyltransferase activity independent manner [7]. In line with this, we observed a slight increase on SFTSV replication in APPA group in Fig 2E. Although these effects are minor compared with that mediated by m^6^A, we still mentioned this possibility and discussed this function of METTL3 in our manuscript (Lines 765-782).

|   New Fig 4J |   Fig 2E |
| --- | --- |

**4)** Related to some of the concerns mentioned above, cell lines with m6A writers (especially METTL3) knockout could be very useful for verifying many results. The KO cell lines could not only be used in the functional studies of Figure 2A but also in the research on drug activity (as shown in Figures 2F and 2G) or site-directed RNA mutations (such as Figures 7F-7J). This could further clarify that the effects of the drug DAA or the impacts caused by RNA sequence mutations are indeed related to methylation modifications. In these KO cells, the effects caused by the drug or mutations are expected to diminish or disappear. These data would provide necessary support for the paper's conclusion.

Thank you for your constructive suggestions. According to your suggestions, we have generated METTL3 knockout (KO) cell lines using CRISPR-Cas9 technology and performed the following experiments:

**1) Functional Studies**: We have repeated the experiments in Figure 2A using METTL3 KO cell lines to verify the impact of m^6^A modification on SFTSV infection (new Fig 2F-2I).

New Fig 2F-2I

**2) Drug Activity:** We have tested the effects of the drug DAA on SFTSV infection in METTL3 KO cells (new Fig 2J).

New Fig 2J

**3) Site-directed RNA mutations**: We also have tested the effects of site-directed RNA mutations on rVSV-SFTSV-G infection in METTL3 KO cells to confirm that the observed effects are indeed related to m^6^A modifications (Fig 7N-7P).

New Fig 7N-7P

We hope these data would further provide necessary support for the paper's conclusion.

**5)** authors preliminarily verified the potential biological significance of G/M RNA methylation on viral infection in vivo using recombinant VSV containing SFTSV G. However, it is unclear whether the modification of G RNA within the VSV genomic circumstance in the context of VSV infection would occur and function in the same manner as during SFTSV infection. Further analyses of the rVSV infections are needed if the authentic challenge with SFTSV cannot be conducted.

Thank you for pointing out this question. For rVSV-SFTSV virus, the only difference is that the m^6^A sites are mutated in the glycoprotein gene of SFTSV, all the other elements are the same. We conducted several experiments to further characterize the difference between the WT and Δm6A virus. As seen in New Fig 7 (new Fig 7D-7F for rSFTSV-G, and new Fig 7N-7P for rVSV-SFTSV-G), the Δm6A viruses are less infective in WT cells, whereas the difference disappeared in M3 KO cells. These data, together with the previous *in vivo* data, furher supported that m^6^A in G gene of SFTSV M segment is important for SFTSV infection. We have put these results in lines 557-561 and lines 612-617.

New Fig 7D-7F

New Fig 7N-7P

**Part III – Minor Issues: Editorial and Data Presentation Modifications**

**1. Reviewer 1**

**1)** The authors should confirm the changes of m6A levels in the cellular or viral genomes when the m6A writers or erasers are depleted or overexpressed.

Thank you for your insightful comment. In response to your suggestion, we performed additional experiments using METTL3 knockout cells. We conducted dot blot assays to assess the m^6^A levels in both cellular total RNA and SFTSV virion RNA. The results showed that m^6^A levels in both cellular total RNA from METTL3 KO cells and SFTSV virion RNA from the supernatants were reduced, confirming the impact of METTL3 depletion on m^6^A modification in both types of RNAs. These new results have been incorporated into the revised manuscript (new Fig 2I).

New Fig 2I

**2)** Fig 6M, the difference in the NP level between WT and mutant viruses is moderate, when compared to viral RNA and titers in 6K/L. The authors may repeat or remove it.

Thank you for your thoughtful comment. As suggested, we have repeated the experiment in Fig 6M to ensure the reliability of the results. The new data confirm the moderate difference in NP levels between WT and mutant viruses. We have updated Fig 6M accordingly.

New Fig 6M

**3)** Although the manuscript is professionally written, it has some grammatical errors and inappropriate wordings. For example, Line 129, is “precipitating” correct in its context? Do the authors mean “ participating” instead? Please proof-read the whole manuscript carefully.

Thank you for your positive feedback. We appreciate your careful review of our manuscript. We have corrected this and thoroughly proofread the entire manuscript to address all grammatical errors and inappropriate wordings. Specific changes include:

1) Line126 Correction: The word "precipitating" has been changed to "accelerating" to accurately reflect the intended meaning.

2) Proofreading: The manuscript has been carefully proofread for grammatical errors, improper word usage, and overall readability. We utilized both automated tools and manual review by multiple team members to ensure accuracy and clarity.

3) Improving Readability: We have refined several sentences to improve their structure and flow, ensuring that the language is precise and the meaning is clear.

**2. Reviewer 2**

**The English writing is generally good, but some portions should be rewritten or revised to make it flows better and easier understood.**

**2.1)** The authors put a lot of information that should be in the main text to help the readers to understand the experimental design into figure legends. For example, the in vivo experiment in mice, a brief description of the experimental design is lacking (Lines 587-589). The readers will have to get this information by digging into the long figure legends.

Thank you for your constructive feedback. As suggested, we have put key information from figure legends into main text (line 619-620) to ensure that readers have easy access to the necessary experimental details without needing to refer to the figure legends..

**2.2)** Line 139, "Current" makes the sentence confusing. It is not clear if the authors referred to their own manuscript or to the reference 41.

Thank you for pointing out the ambiguity in the sentence. To clarify, the term "current" in the original text was intended to refer to the general state of research in the field, not specifically to our own manuscript or reference 41. To improve clarity, I have revised the sentence to more explicitly convey this meaning (line 136).

**2.3) "RNA's" modification or "m6A's ability" have been used in few places. It will read better as modification of the viral RNA, as such.**

Thank you for your valuable feedback. I have revised the manuscript to address the issue with the phrasing. Specifically, I have replaced "RNA's modification" and "m^6^A's ability" with "the modification of the viral RNA" and similar terminology throughout the manuscript in the relevant sections, such as lines 31, 49, 712, 794, 802-803, and so on. I appreciate your input in improving the clarity of our manuscript.

**3. Reviewer 3**

**3.1)** Fig S1: The methylation modification patterns observed in different cells vary significantly. Please provide further analysis or discussion.

Thank you for your feedback regarding the distict methylation modification patterns observed in different cells. Our results indicate significant variabilities in m^6^A methylations between different cell types and between intracellular and virion-associated RNA. Specifically, the similarity in m^6^A patterns in S/M genomic RNA suggests a selective packaging of modified RNA into virions, which may be crucial for viral replication or immune evasion. Conversely, the observed differences in L genomic RNA m^6^A modifications could be attributed to distinct packaging strategies or host-specific factors. We have provided further analysis in the results section (lines 188-196) and incorporated this comments in the discussion section (lines 727-734).

**3.2)** Fig 3: The changes in protein expression are minimal and inconsistent. Overall, this result is relatively weak and could be considered for further analysis or placed in supplementary materials.

Thank you for your observation regarding Fig 3. We acknowledge that the changes in protein expression for METTL3 and METTL14 may appear modest. We have performed western blot analysis on Huh7 cells infected with SFTSV at different time points, and showed upregulation of both METTL3 and METTL14 proteins, which is accompanied by an increase in m^6^A levels in total RNA as confirmed by Dot blot analysis. While the degree in protein upregulation may not be markedly different, they are reproducible and align with the m^6^A level increases we observed during SFTSV infection, suggesting a biologically relevant role in this context. We have updated the manuscript accordingly (lines 302-304) and included these new data in new Fig 3D.

Additionally, we are open to placing this figure in the supplementary materials, although we believe it provides important insights into the role of METTL3 and m^6^A during SFTSV infection. We hope these clarifications and additional analyses adequately address your concerns.

New Fig 3D

**3.3)** Fig 6: Are the predicted methylation patterns consistent with those actually identified? Why not refer to the results obtained from sequencing identification? How were the viruses packaged using reverse genetics amplified to high titers, and were the mutants sequenced for verification? Please provide more details necessary here.

Thank you for your insightful comments regarding Fig 6 and the experimental approach. The m^6^A sites we selected for validation were not all derived exclusively from enriched regions identified in the MeRIP-seq results. Since MeRIP-seq does not provide single-base resolution, we used a combination of m^6^A motif prediction and our MeRIP-seq data to identify potential methylation sites. Specifically, we predicted m^6^A sites using the SRAMP database [8] based on established consensus motifs, cross-referenced them with enriched peaks from our MeRIP-seq data, and employed dCas13b-YTHDF2 with gRNAs that target different m^6^A sites, as described before [9], to select the most likely sites for further validation. This integrated approach allowed us to determine several potential m^6^A sites, and we mutated these consensus sequences to create the m^6^A mutant viruses.

To amplify the recombinant viruses (rSFTSVs or rVSVs) to high titers, we took similar approaches according to previously published reports [10, 11] (lines 1220-1236). we conducted multiple rounds of virus production across several batches. Ultimately, we concentrated the viruses using sucrose gradient centrifugation to achieve the desired viral titers. The viral mutants were subjected to sequencing verification to confirm the presence of the intended mutations (Fig 6I and Fig 7C).

**3.4)** In this study, how were the mRNAs of viral structural proteins and antigenome RNAs, as well as the NSs mRNA and S genome RNA, distinguished?

Thank you for your question. The S segment utilizes an ambi-sense coding strategy, where the N mRNA is transcribed from the genomic RNA, while the NSs mRNA is transcribed from the antigenomic RNA. Due to the overlapping nature of these sequences, distinguishing between the NP mRNA and antigenome RNA, as well as between the NSs mRNA and S genome RNA, presents a challenge during infection.

Since the S antigenome also contains the NP mRNA sequence, and the S genome contains the NSs mRNA sequence, it is difficult to accurately differentiate these RNA species under normal infection conditions.

To overcome this, we co-transfected NP and METTL3-encoding plasmids into HEK293T cells in a controlled setting, which allowed us to isolate the expression of viral proteins from the influence of viral genomic or antigenomic RNA. This approach enabled us to focus on the effects of METTL3 on viral protein expression without interference from the overlapping RNA species. We have put this information in the result section (line 412-415) and in the discussion section (lines 789-792).

**3.5)** Fig 8A and 8B: The colors used for the two proteins are too similar; please use more contrasting colors to label different proteins.

**A:**Thank you for your valuable feedback regarding the color scheme in Fig 8A and 8B. As suggested, we have modified the figure and now use blue and red to distinguish the two proteins.

New Fig 8A-8B

**3.6)** line 34-35, please specify the use of surrogate viruses, rVSV.

**A:** Thank you for your insightful comment. We apologize for the lack of clarity in the original text. To address your concern, we have now specified the use of recombinant vesicular stomatitis virus (rVSV) as a surrogate virus. The revised sentence in lines 34-35.

**3.7)** line 95 and the following, METTL3 were written as **METTTL**3.

Thank you for pointing out the typo in "METTTL3" in line 95 and the following. We have corrected it to "METTL3" in line 94, 95, and 97. We appreciate your careful review and attention to detail.

We are grateful to the reviewers for their valuable remarks and do hope that the revised manuscript is can be reconsidered for publication in *PLOS Pathogens*.

Sincerely yours,

Jianfeng Dai, Ph.D.

Professor of Microbiology and Immunology

1.	Tercero B, Narayanan K, Terasaki K, Makino S. Characterization of the Molecular Interactions That Govern the Packaging of Viral RNA Segments into Rift Valley Fever Phlebovirus Particles. J Virol. 2021;95(14):e0042921. Epub 2021/05/07. doi: 10.1128/JVI.00429-21. PubMed PMID: 33952635; PubMed Central PMCID: PMCPMC8223960.

2.	Tercero B, Terasaki K, Narayanan K, Makino S. Mechanistic insight into the efficient packaging of antigenomic S RNA into Rift Valley fever virus particles. Front Cell Infect Microbiol. 2023;13:1132757. Epub 20230216. doi: 10.3389/fcimb.2023.1132757. PubMed PMID: 36875526; PubMed Central PMCID: PMCPMC9978001.

3.	Xue M, Zhao BS, Zhang Z, Lu M, Harder O, Chen P, et al. Viral N(6)-methyladenosine upregulates replication and pathogenesis of human respiratory syncytial virus. Nat Commun. 2019;10(1):4595. Epub 2019/10/11. doi: 10.1038/s41467-019-12504-y. PubMed PMID: 31597913; PubMed Central PMCID: PMCPMC6785563.

4.	Lu M, Zhang Z, Xue M, Zhao BS, Harder O, Li A, et al. N(6)-methyladenosine modification enables viral RNA to escape recognition by RNA sensor RIG-I. Nat Microbiol. 2020;5(4):584-98. Epub 2020/02/06. doi: 10.1038/s41564-019-0653-9. PubMed PMID: 32015498; PubMed Central PMCID: PMCPMC7137398.

5.	Lu M, Xue M, Wang HT, Kairis EL, Ahmad S, Wei J, et al. Nonsegmented Negative-Sense RNA Viruses Utilize N(6)-Methyladenosine (m(6)A) as a Common Strategy To Evade Host Innate Immunity. J Virol. 2021;95(9). Epub 2021/02/05. doi: 10.1128/JVI.01939-20. PubMed PMID: 33536170; PubMed Central PMCID: PMCPMC8104112.

6.	Hao H, Hao S, Chen H, Chen Z, Zhang Y, Wang J, et al. N6-methyladenosine modification and METTL3 modulate enterovirus 71 replication. Nucleic Acids Res. 2019;47(1):362-74. Epub 2018/10/27. doi: 10.1093/nar/gky1007. PubMed PMID: 30364964; PubMed Central PMCID: PMCPMC6326802.

7.	Wei X, Huo Y, Pi J, Gao Y, Rao S, He M, et al. METTL3 preferentially enhances non-m(6)A translation of epigenetic factors and promotes tumourigenesis. Nat Cell Biol. 2022;24(8):1278-90. Epub 2022/08/05. doi: 10.1038/s41556-022-00968-y. PubMed PMID: 35927451.

8.	Zhou Y, Zeng P, Li YH, Zhang Z, Cui Q. SRAMP: prediction of mammalian N6-methyladenosine (m6A) sites based on sequence-derived features. Nucleic Acids Res. 2016;44(10):e91. Epub 2016/02/21. doi: 10.1093/nar/gkw104. PubMed PMID: 26896799; PubMed Central PMCID: PMCPMC4889921.

9.	Rauch S, He C, Dickinson BC. Targeted m(6)A Reader Proteins To Study Epitranscriptomic Regulation of Single RNAs. J Am Chem Soc. 2018;140(38):11974-81. Epub 2018/09/06. doi: 10.1021/jacs.8b05012. PubMed PMID: 30183280; PubMed Central PMCID: PMCPMC6436614.

10.	Brennan B, Li P, Zhang S, Li A, Liang M, Li D, Elliott RM. Reverse genetics system for severe fever with thrombocytopenia syndrome virus. J Virol. 2015;89(6):3026-37. Epub 2015/01/02. doi: 10.1128/JVI.03432-14. PubMed PMID: 25552716; PubMed Central PMCID: PMCPMC4337530.

11.	Xu M, Wang B, Deng F, Wang H, Wang M, Hu Z, Liu J. Establishment of a Reverse Genetic System of Severe Fever with Thrombocytopenia Syndrome Virus Based on a C4 Strain. Virol Sin. 2021;36(5):958-67. Epub 2021/03/16. doi: 10.1007/s12250-021-00359-x. PubMed PMID: 33721215; PubMed Central PMCID: PMCPMC8558135.
